# Supplementary material for: Improved Post-Thaw Quality of Canine Semen after Treatment with Exosomes from Conditioned Medium of Adipose-Derived Mesenchymal Stem Cells
Source: Animals (Basel). 2019 Oct 25;9(11):865. doi: 10.3390/ani9110865 (PMC6912283; doi:10.3390/ani9110865)
Supplement: Supplementary file 1 [file animals-09-00865-s001.pdf]

animals

# Supplementary Materials: Improved Post-Thaw Quality of Canine Semen after Treatment with Exosomes from Conditioned Medium of Adipose-Derived Mesenchymal Stem Cells

**Ahmad Yar Qamar [1], Xun Fang [1], Min Jung Kim [2,*] and Jongki Cho [1,*]**

[1] College of Veterinary Medicine, Chungnam National University, Daejeon 34134, Korea; drahmadqamar@gmail.com (A.Y.Q.); fx2442@gmail.com (X.F.)
[2] Department of Theriogenology and Biotechnology, College of Veterinary Medicine, Seoul National University, Seoul 08826, Korea
* Correspondence: tinia19@snu.ac.kr (M.J.K.); cjki@cnu.ac.kr (J.C.); Tel.: +82-42-821-6788 (J.C.) Fax: +82-42-821-8903 (J.C.)

**Table S1.** Motility characteristics of dog sperm cooled at 4 °C for 8–10 h.

| Groups | Progressive motility (%) | VCL (μm/sec) | VSL (μm/sec) | VAP (μm/sec) | BCF (Hz) |
|---|---|---|---|---|---|
| Control | 40.5 ± 0.4 [a,b,c] | 98.5 ± 0.8 [b] | 31.1 ± 0.6 | 50.9 ± 0.6 | 5.5 ± 0.2 |
| 25 μg/ mL | 39.5 ± 0.3 [b] | 96.0 ± 1.1 [b] | 29.9 ± 0.6 | 49.0 ± 0.6 | 5.4 ± 0.2 |
| 50 μg/ mL | 41.6 ± 0.7 [a] | 103.1 ± 1.1 [a] | 31.4 ± 0.3 | 52.7 ± 0.8 | 6.1 ± 0.3 |
| 100 μg/ mL | 38.6 ± 0.4 [c] | 96.8 ± 1.1 [b] | 29.8 ± 0.5 | 50.2 ± 0.6 | 5.4 ± 0.6 |

Values with different superscripts letters in a column differ significantly ($p$ < 0.05, $n$ = 4). VCL, Curvilinear velocity; VSL, Straight-line velocity; VAP, Average path velocity; BCF, Beat cross frequency.

**Table S2.** Effect of exosomal treatment on the progressive motility and velocities of post-thaw dog semen.

| Group | Progressive motility (%) | VCL (μm/sec) | VSL (μm/sec) | VAP (μm/sec) | BCF (Hz) |
|---|---|---|---|---|---|
| Control | 20.9 ± 0.6 [b] | 50.1 ± 0.9 [b] | 17.7 ± 0.7 [b] | 27.7 ± 0.3 [b] | 2.5 ± 0.0 [b] |
| Treatment (50 μg/mL) | 25.3 ± 0.8 [a] | 62.6 ± 1.1 [a] | 20.6 ± 0.5 [a] | 32.1 ± 0.5 [a] | 3.2 ± 0.1 [a] |

Values with different superscripts letters in a column differ significantly ($p$ < 0.05, $n$ = 4). VCL, Curvilinear velocity; VSL, Straight-line velocity; VAP, Average path velocity; BCF, Beat cross frequency.
